# Clinical Utility of 18F-Fluorodeoxyglucose Positron Emission Tomography/Computed Tomography (FDG-PET/CT) in Suspected Spondylodiscitis: A Case Report

**DOI:** 10.7759/cureus.103492

**Published:** 2026-02-12

**Authors:** David Gutierrez Albenda, Ernesto Balmaceda Araya, Valeria Armani Arce, Akira Osawa Pivovarov, Álvaro Montoya Porras

**Affiliations:** 1 Cyclotron-PET/CT Laboratory, University of Costa Rica, San José, CRI; 2 School of Medicine, University of Costa Rica, San José, CRI

**Keywords:** 18f fdg-pet/ct, clinical case report, nuclear medicine imaging, spinal instrumentation surgery, spondylodiscitis

## Abstract

Nuclear medicine has undergone remarkable advances in recent decades. Among these, 18F-fluorodeoxyglucose positron emission tomography/computed tomography (FDG-PET/CT) has become a key imaging modality, enabling metabolic assessment of tissues and precise anatomical correlation, thereby improving diagnostic accuracy.

We present the case of a 64-year-old woman with fever, pain, and a deteriorated general condition, initially suspected of having a hip prosthesis infection. FDG-PET/CT imaging played a pivotal role in suggesting the diagnosis of spondylodiscitis. We also discuss the relevance of this modality, its broad range of current clinical applications, and its specific utility in diagnosing and managing spinal infections.

## Introduction

Nuclear medicine imaging has become an essential tool in modern medical practice. Among these techniques, 18F-fluorodeoxyglucose positron emission tomography/computed tomography (FDG-PET/CT) employs a radiotracer to delineate metabolic activity across different body regions [1]. The radiotracer used, fluorodeoxyglucose (FDG), is a glucose analog that accumulates in tissues with increased cellular metabolism, such as those affected by oncologic, infectious, or inflammatory processes [2].

The first study using FDG was published in 1976 [3], demonstrating physiologic uptake in organs such as the brain, heart, and urinary bladder [4,5]. Since then, the technique has evolved and now has extensive clinical applications, including metastatic disease assessment, fever of unknown origin, and autoimmune or vasculitic processes [6]. FDG offers high sensitivity, high-resolution imaging, and minimal adverse effects [7].

Spondylodiscitis, an infectious process involving the intervertebral disc and adjacent vertebral endplates [8], can result from hematogenous spread or direct inoculation, often as a postoperative complication. It is a diagnostic challenge because it presents with nonspecific, slowly progressive symptoms such as back pain, often without fever or marked laboratory abnormalities. Diagnosis is further complicated by delayed or inconclusive imaging findings and frequent confounders such as degenerative changes, malignancy, or postoperative alterations. The most common pathogens are Staphylococcus aureus, Gram-negative bacilli, and streptococci, though Brucella and Mycobacterium tuberculosis have also been reported [9,10].

While magnetic resonance imaging (MRI) remains the gold standard for diagnosis, FDG-PET/CT is particularly useful when MRI findings are inconclusive due to metallic artifacts, postoperative changes, or difficulty differentiating degenerative from infectious processes. Additionally, PET/CT allows whole-body assessment, facilitating detection of multifocal infection and alternative infectious foci [11]. Our objective with this case report is to demonstrate its use in a case with the aforementioned limitations and show its impact on clinical decision-making.

## Case presentation

A 64-year-old woman with a medical history of cervical cancer, lumbar arthrodesis with parallel rods from L2 to S1 (2021), and total right hip replacement following a fracture (2022) presented to the emergency department with joint pain, fever, functional limitation, and general malaise. Laboratory tests revealed elevated C-reactive protein and thrombocytosis, without leukocytosis.

An acute prosthetic joint infection was initially suspected, and empirical vancomycin therapy was initiated, with partial clinical improvement. Hip arthrocentesis yielded negative aerobic and anaerobic cultures. An MRI was performed at the referring institution and was described in the referral documentation as showing nonspecific postoperative and degenerative changes, without definitive signs of infection. As our institution functions as a regional reference center for diagnostic imaging, we did not have direct access to the MRI images or the full report, which precluded their inclusion in this case report.

**Figure 1:**
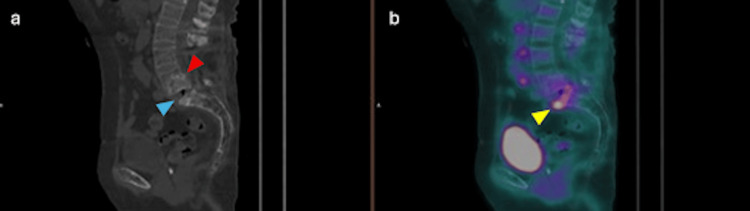
(a) Sagittal CT of the lumbosacral spine showing partial L4–L5 fusion (red arrowhead), and sclerotic/erosive changes at L5–S1 (blue arrowhead). (b) Sagittal 18F-FDG PET/CT showing intense hypermetabolism corresponding to the L5-S1 CT changes (yellow arrowhead), extending into the anterior soft tissues.

Due to persistently elevated inflammatory markers and uncertainty about the infectious focus, FDG-PET/CT was performed to distinguish between a delayed prosthetic joint infection and a spinal implant-related process. The scan (Figure 1) revealed retroperitoneal lymphadenopathy in the interaortocaval and retroaortic regions, which was interpreted as most likely reactive in accordance with the other findings (SUVmax: 4.9). Partial fusion changes were noted at L4-L5, with L4-over-L5 spondylolisthesis, likely inflammatory (SUVmax: 4.4). Intense hypermetabolism was observed at the inferior endplate of L5 and the superior endplate of S1, with associated sclerotic and erosive changes and extension into the anterior soft tissues. These findings are suggestive of spondylodiscitis at the L5-S1 level, with extension into the adjacent soft tissues and probable associated osteomyelitis.

**Figure 2:**
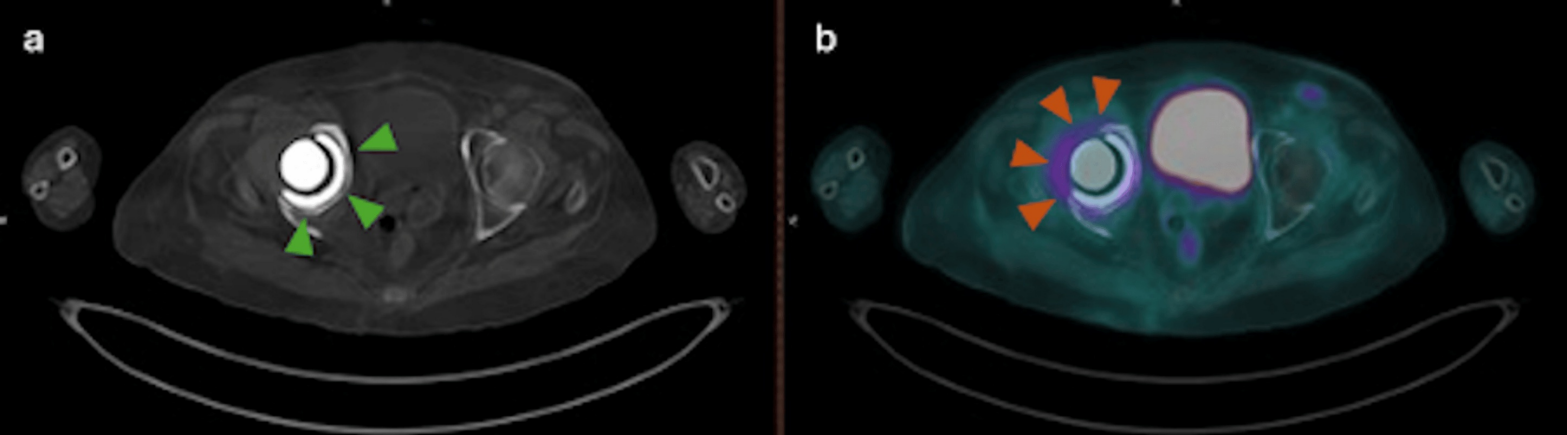
(a) Axial CT of the pelvis showing the right hip prosthesis (green arrowheads). (b) Axial 18F-FDG PET showing increased uptake around the prosthesis (orange arrowheads), consistent with inflammatory rather than infectious activity.

Increased peri-prosthetic FDG uptake surrounding the right hip replacement (Figure 2) was interpreted as inflammatory rather than infectious in nature (SUVmax: 4.2). No validated SUV cutoff exists to reliably distinguish infection from inflammation; therefore, interpretation was based on uptake pattern, distribution, and correlation with CT findings and clinical context, which in this case favored an inflammatory process. The complete report was communicated to the treating team for further management planning. 

## Discussion

Given the patient’s symptoms, an initial diagnosis of prosthetic hip infection was reasonable. However, subsequent imaging clarified that the pathology was spinal in origin. This presentation, fever, malaise, and elevated inflammatory markers, is typical of spondylodiscitis, although its nonspecificity often delays diagnosis [12].

Imaging is essential to confirm the diagnosis. MRI remains the first-line modality for its high sensitivity and specificity, yet its role in follow-up is limited, and results may be inconclusive in some cases, like metal instrumentation or multifocal infection [13]. FDG-PET/CT provides superior differentiation between degenerative and infectious/inflammatory processes and is valuable for monitoring disease progression [14,15].

Treatment typically begins with aggressive intravenous antibiotic therapy [16]. Surgical intervention may be warranted in cases of neurological compromise, abscess formation, or significant structural damage [17]. Minimally invasive or endoscopic approaches have also shown promising results [18].

Despite its diagnostic utility, FDG-PET/CT remains underutilized in many regions, particularly in Latin America [19]. Beyond bacterial infections, this modality can detect inflammatory activity associated with viral or mycobacterial infections [20]. The development of cost-effective systems may help expand its accessibility in resource-limited settings.

## Conclusions

Spondylodiscitis poses a considerable diagnostic challenge due to its nonspecific clinical presentation. MRI remains the first-line imaging modality for suspected spondylodiscitis. In cases like this one, where conventional imaging is limited by extensive spinal instrumentation and confounded by potential distractors such as a hip prosthesis, FDG-PET/CT proves to be an invaluable diagnostic tool. Its value extends beyond spinal infections, as it is also highly effective in evaluating prosthetic and other musculoskeletal infections.

The unique ability of FDG-PET/CT to differentiate active infection, assess disease extent, and monitor treatment response underscores its expanding role in precision medicine. Expanding access to this technology should be an important technological advancement priority, particularly in developing healthcare systems that strive for better precision in testing.

## References

[1] Basu S, Hess S, Nielsen Braad PE, Olsen BB, Inglev S, Høilund-Carlsen PF (2014). The basic principles of FDG-PET/CT imaging. PET Clin.

[2] Litt HK, Kwon DH, Velazquez AI (2023). FDG PET scans in cancer care. JAMA Oncol.

[3] Hess S, Høilund-Carlsen PF, Alavi A (2014). Historic images in nuclear medicine: 1976: the first issue of clinical nuclear medicine and the first human FDG study. Clin Nucl Med.

[4] Bacchiani M, Salamone V, Massaro E (2023). Assessing the performance of 18F-FDG PET/CT in bladder cancer: a narrative review of current evidence. Cancers (Basel).

[5] Wachsmann JW, Gerbaudo VH (2014). Thorax: normal and benign pathologic patterns in FDG-PET/CT imaging. PET Clin.

[6] Kobayashi T, Miyamori D, Ito M (2024). Retrospective study on clinical value and optimal use of [(18)F] FDG PET/CT for inflammation of unknown origin in Japanese patients. Sci Rep.

[7] Hess S, Hansson SH, Pedersen KT, Basu S, Høilund-Carlsen PF (2014). FDG-PET/CT in infectious and inflammatory diseases. PET Clin.

[8] Emilie S, Zeller V, Fautrel B, Aubry A (2016). Spondylodiscitis (Article in Spanish). EMC - Tratado de Medicina.

[9] Couderc M, Tournadre A, Soubrier M, Dubost J-J (2022). Pathology of the spine: nontuberculous infectious spondylodiscitis (Article in Spanish). EMC - Aparato Locomotor.

[10] Sade R, Polat G, Ogul H, Kantarci M (2017). Brucella spondylodiscitis. Med Clin (Barc).

[11] Noriega-Álvarez E, Rodríguez Alfonso B, Rosales Castillo JJ (2025). Role and applications of 18F-FDG PET/CT in the assessment of osteoarticular infection and inflammation - part I (Article in Spanish). Revista Española de Medicina Nuclear e Imagen Molecular.

[12] Altabás MA, Altabás MJ (2023). Spondylodiscitis: a diagnostic challenge. Visual J Emerg Med.

[13] Raghavan M, Palestro CJ (2023). Imaging of spondylodiscitis: an update. Semin Nucl Med.

[14] Noriega-Álvarez E, Domínguez Gadea L, Orduña Diez MP, Peiró Valgañón V, Sanz Viedma S, García Jiménez R (2019). Role of nuclear medicine in the diagnosis of musculoskeletal infection: a review. Rev Esp Med Nucl Imagen Mol (Engl Ed).

[15] Smids C, Kouijzer IJ, Vos FJ (2017). A comparison of the diagnostic value of MRI and (18)F-FDG-PET/CT in suspected spondylodiscitis. Infection.

[16] Livorsi DJ, Daver NG, Atmar RL, Shelburne SA, White AC Jr, Musher DM (2008). Outcomes of treatment for hematogenous Staphylococcus aureus vertebral osteomyelitis in the MRSA ERA. J Infect.

[17] Martín-Alonso J, Delgado-López PD, Castilla-Díez JM (2018). Role of surgery in spontaneous spondylodiscitis: experience in 83 consecutive patients (Article in Spanish). Neurocirugia (Engl Ed).

[18] Abreu PG, Lourenço JA, Romero C (2022). Endoscopic treatment of spondylodiscitis: systematic review. Eur Spine J.

[19] Kwee TC, Basu S, Alavi A (2015). Should the nuclear medicine community continue to underestimate the potential of 18F-FDG-PET/CT with present generation scanners for the diagnosis of prosthetic joint infection?. Nucl Med Commun.

[20] Kleynhans J, Sathekge MM, Ebenhan T (2023). Preclinical research highlighting contemporary targeting mechanisms of radiolabelled compounds for PET based infection imaging. Semin Nucl Med.

